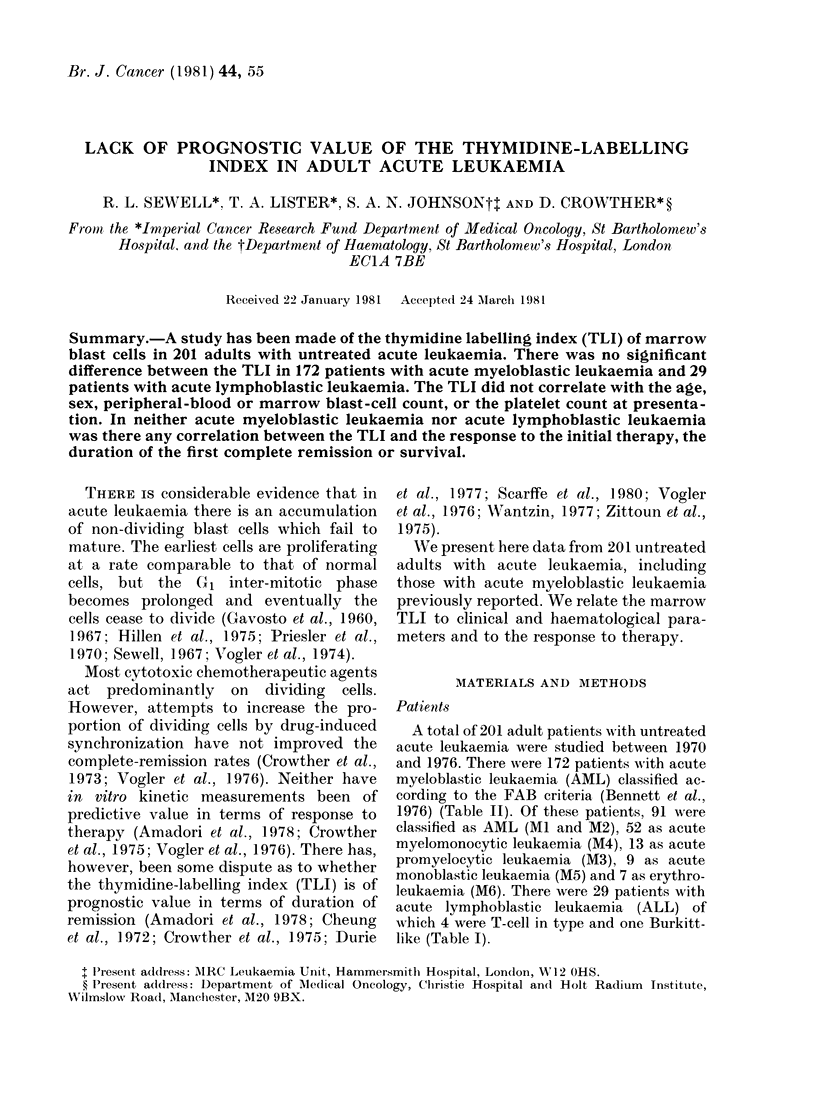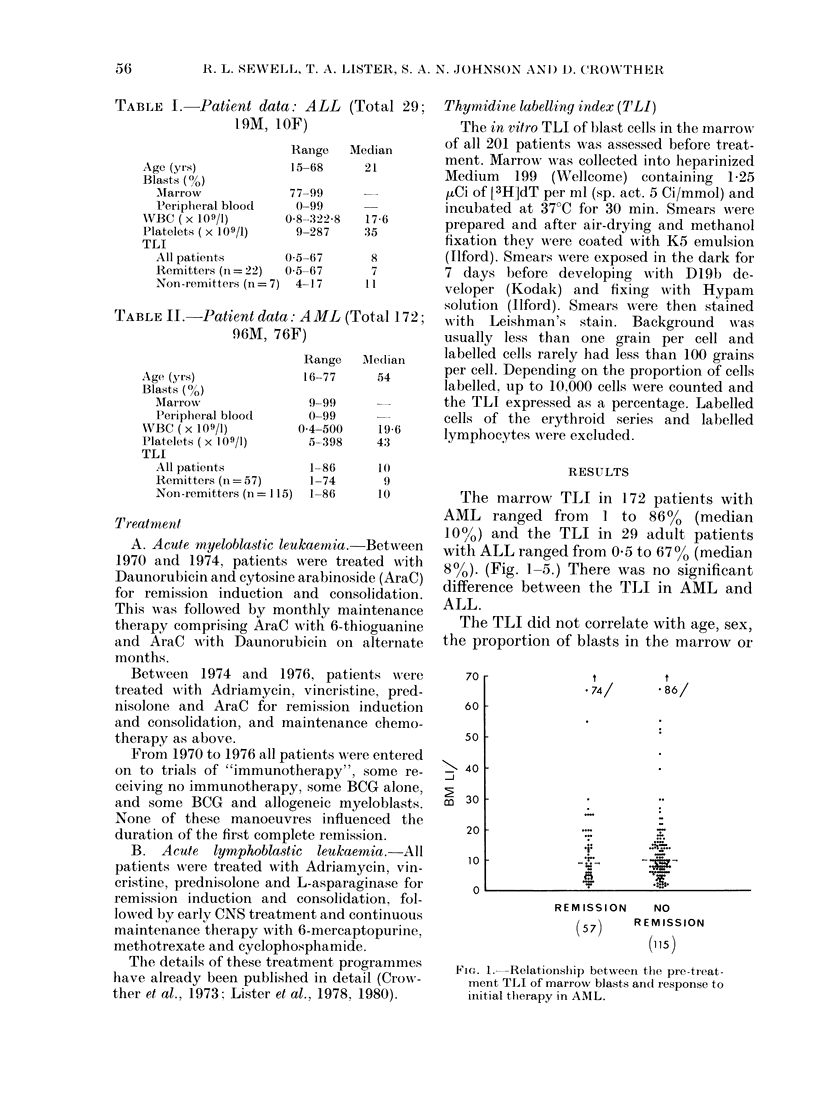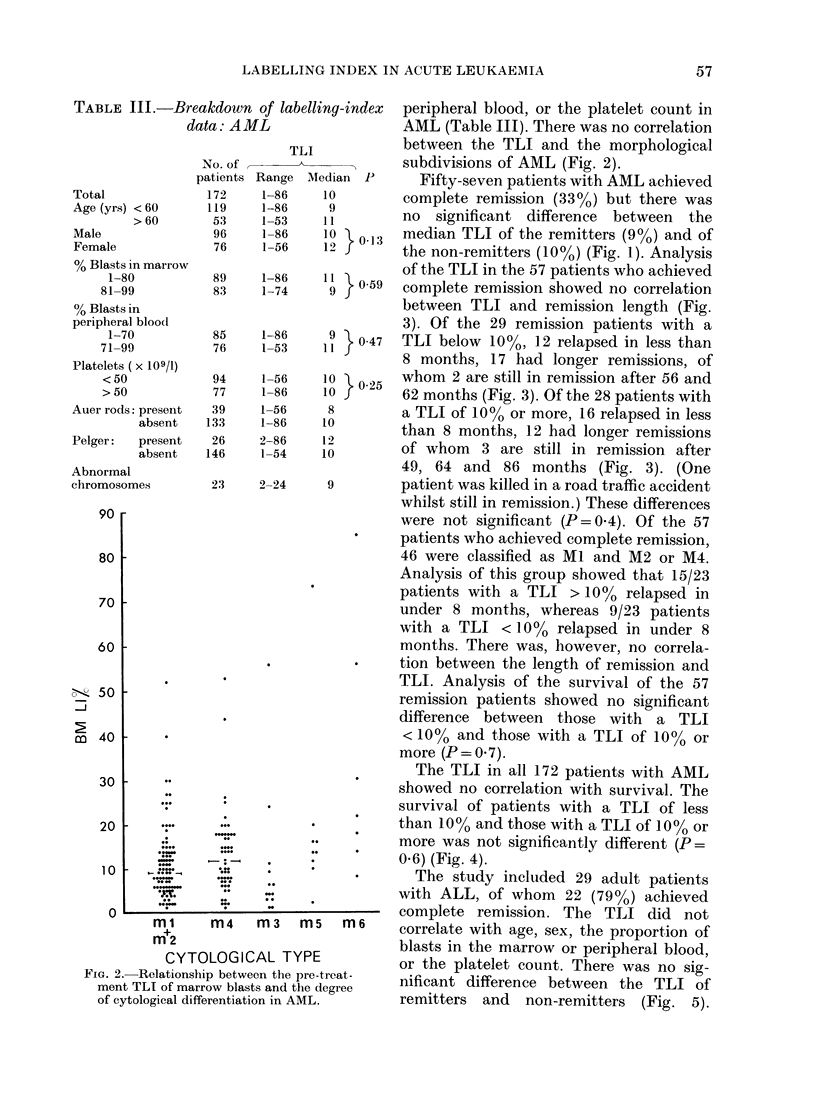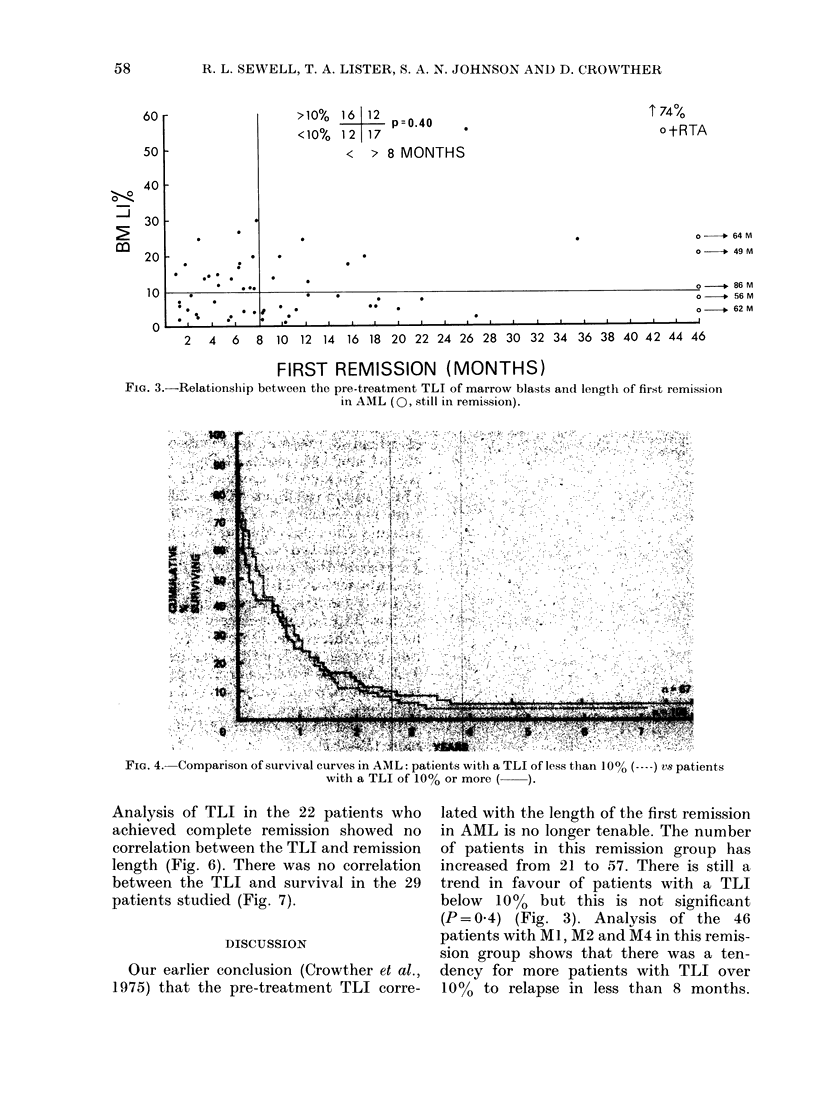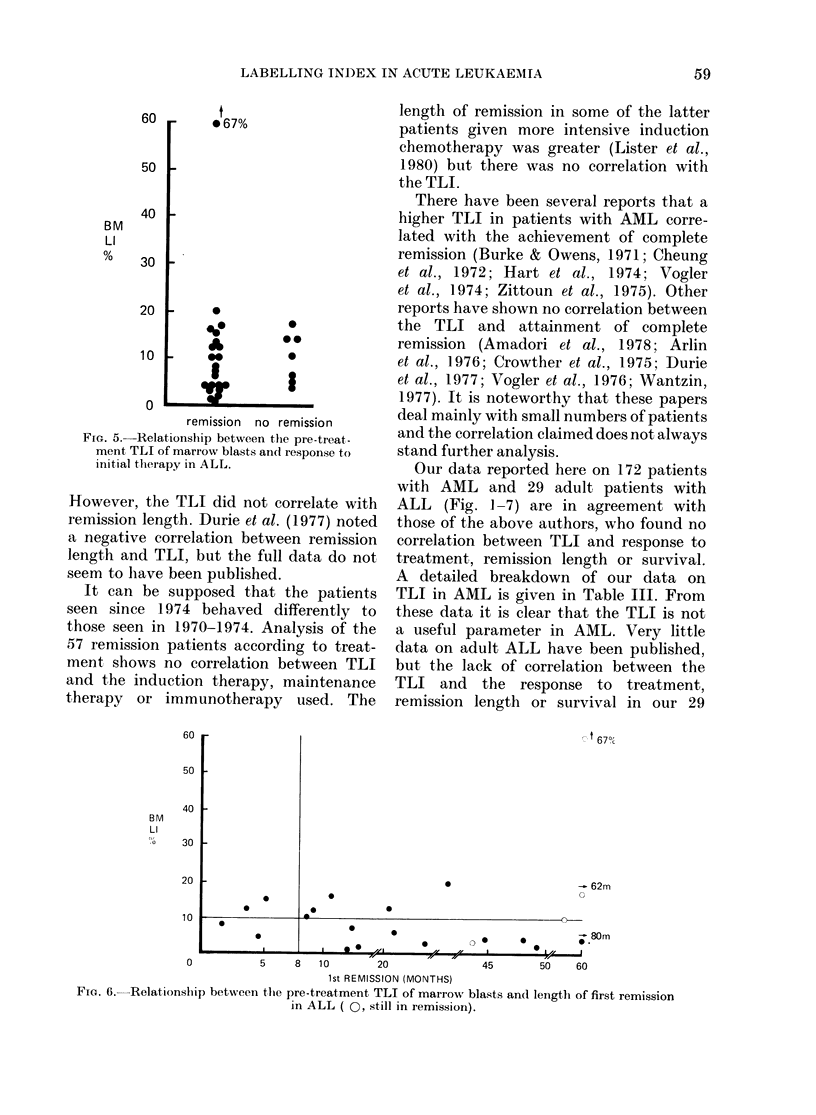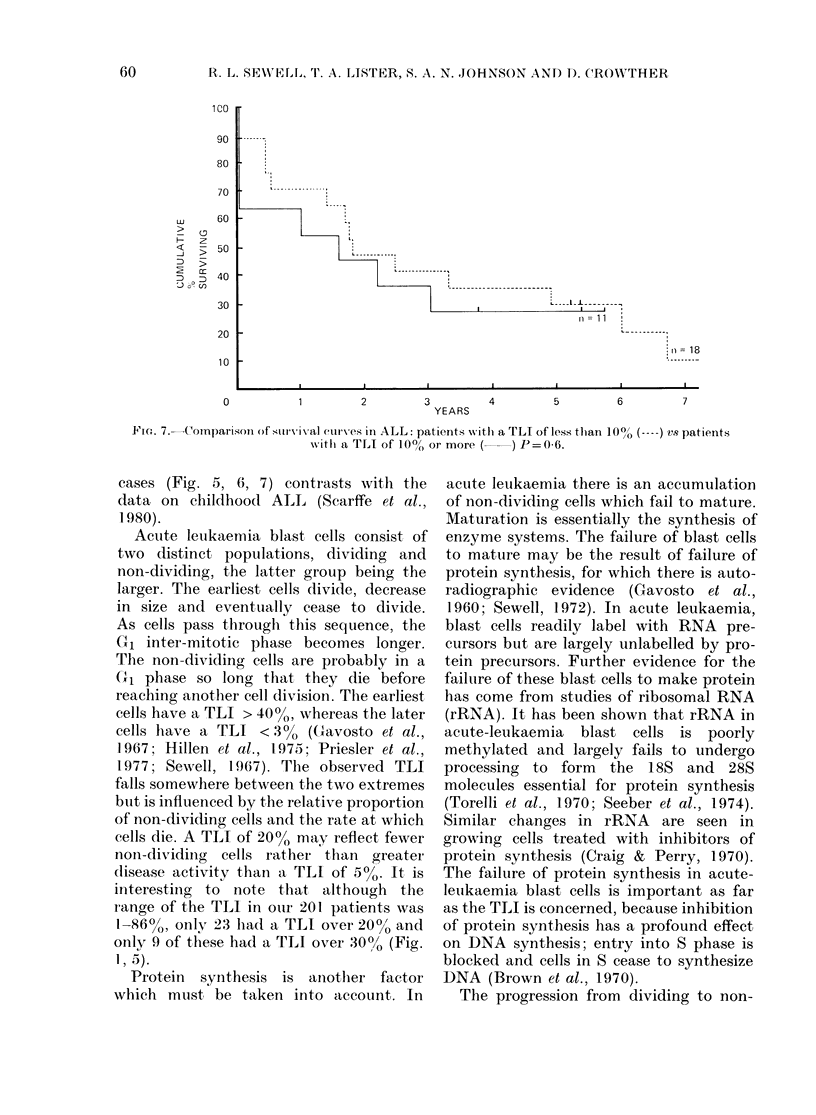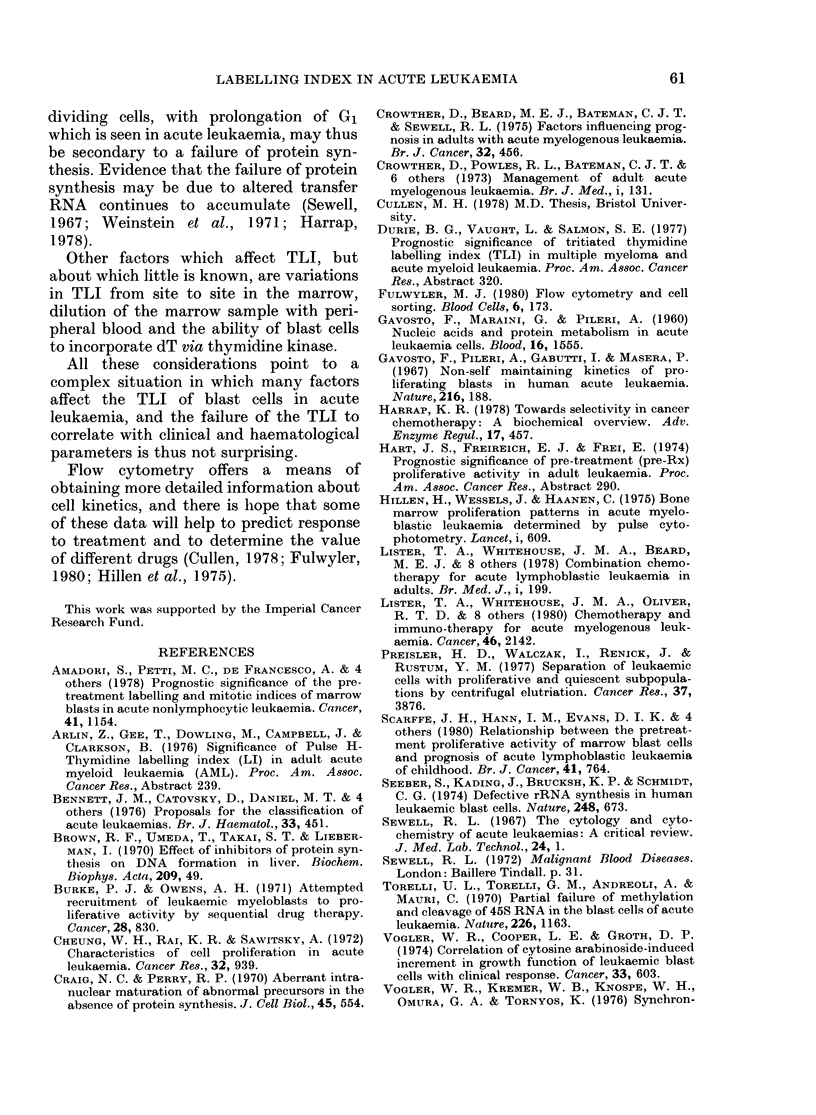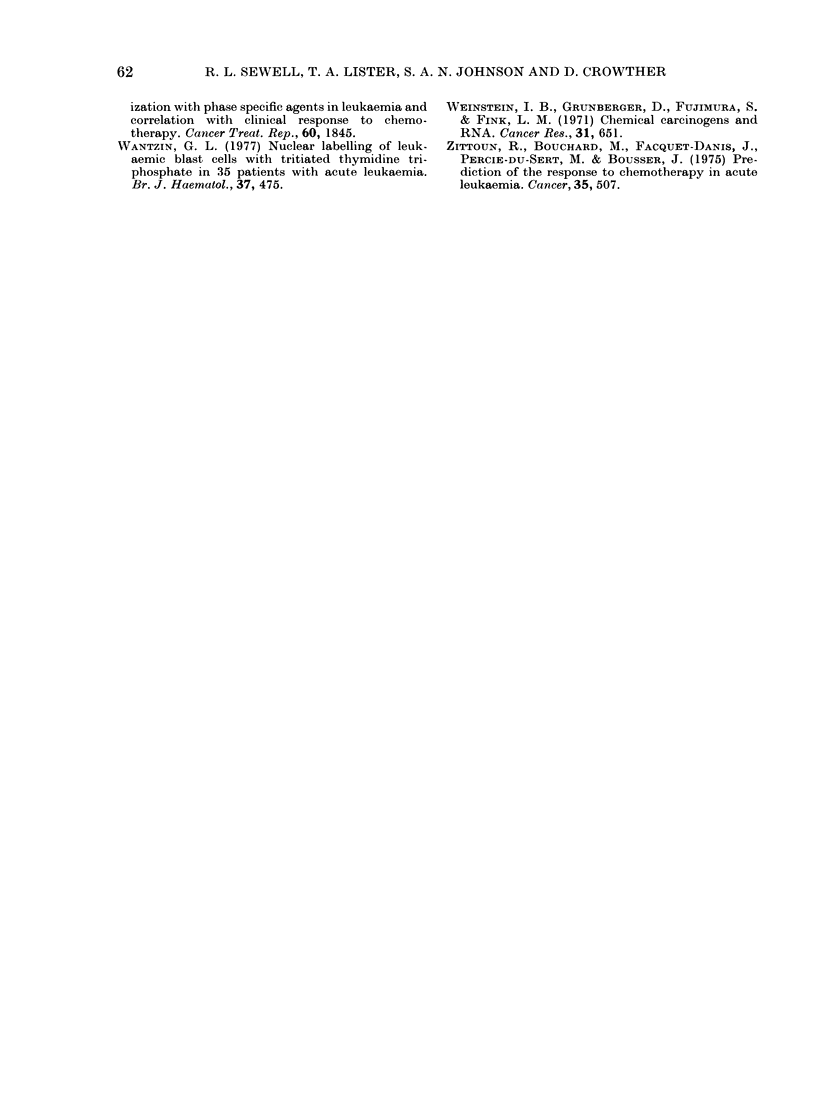# Lack of prognostic value of the thymidine-labelling index in adult acute leukaemia.

**DOI:** 10.1038/bjc.1981.147

**Published:** 1981-07

**Authors:** R. L. Sewell, T. A. Lister, S. A. Johnson, D. Crowther

## Abstract

A study has been made of the thymidine labelling index (TLI) of marrow blast cells in 201 adults with untreated acute leukaemia. There was no significant difference between the TLI in 172 patients with acute myeloblastic leukaemia and 29 patients with acute lymphoblastic leukaemia. The TLI did not correlate with the age, sex, peripheral-blood or marrow blast-cell count, or the platelet count at presentation. In neither acute myeloblastic leukaemia nor acute lymphoblastic leukaemia was there any correlation between the TLI and the response to the initial therapy, the duration of the first complete remission or survival.


*Br. J. Cancer* (1981) **44**, 55

### LACK OF PROGNOSTIC VALUE OF THE THYMIDINE-LABELLING INDEX IN ADULT ACUTE LEUKAEMIA

R. L. SEWELL*, T. A. LISTER*, S. A. N. JOHNSON†‡ AND D. CROWTHER*§

*From the *Imperial Cancer Research Fund Department of Medical Oncology, St Bartholomew's Hospital, and the †Department of Haematology, St Bartholomew's Hospital, London EC1A 7BE*

Received 22 January 1981 Accepted 24 March 1981

**Summary.—A study has been made of the thymidine labelling index (TLI) of marrow blast cells in 201 adults with untreated acute leukaemia. There was no significant difference between the TLI in 172 patients with acute myeloblastic leukaemia and 29 patients with acute lymphoblastic leukaemia. The TLI did not correlate with the age, sex, peripheral-blood or marrow blast-cell count, or the platelet count at presentation. In neither acute myeloblastic leukaemia nor acute lymphoblastic leukaemia was there any correlation between the TLI and the response to the initial therapy, the duration of the first complete remission or survival.**

THERE IS considerable evidence that in acute leukaemia there is an accumulation of non-dividing blast cells which fail to mature. The earliest cells are proliferating at a rate comparable to that of normal cells, but the $G_1$ inter-mitotic phase becomes prolonged and eventually the cells cease to divide (Gavosto *et al.*, 1960, 1967; Hillen *et al.*, 1975; Priesler *et al.*, 1970; Sewell, 1967; Vogler *et al.*, 1974).

Most cytotoxic chemotherapeutic agents act predominantly on dividing cells. However, attempts to increase the proportion of dividing cells by drug-induced synchronization have not improved the complete-remission rates (Crowther *et al.*, 1973; Vogler *et al.*, 1976). Neither have *in vitro* kinetic measurements been of predictive value in terms of response to therapy (Amadori *et al.*, 1978; Crowther *et al.*, 1975; Vogler *et al.*, 1976). There has, however, been some dispute as to whether the thymidine-labelling index (TLI) is of prognostic value in terms of duration of remission (Amadori *et al.*, 1978; Cheung *et al.*, 1972; Crowther *et al.*, 1975; Durie *et al.*, 1977; Scarffe *et al.*, 1980; Vogler *et al.*, 1976; Wantzin, 1977; Zittoun *et al.*, 1975).

We present here data from 201 untreated adults with acute leukaemia, including those with acute myeloblastic leukaemia previously reported. We relate the marrow TLI to clinical and haematological parameters and to the response to therapy.

### MATERIALS AND METHODS

*Patients*

A total of 201 adult patients with untreated acute leukaemia were studied between 1970 and 1976. There were 172 patients with acute myeloblastic leukaemia (AML) classified according to the FAB criteria (Bennett *et al.*, 1976) (Table II). Of these patients, 91 were classified as AML (M1 and M2), 52 as acute myelomonocytic leukaemia (M4), 13 as acute promyelocytic leukaemia (M3), 9 as acute monoblastic leukaemia (M5) and 7 as erythroleukaemia (M6). There were 29 patients with acute lymphoblastic leukaemia (ALL) of which 4 were T-cell in type and one Burkitt-like (Table I).

‡ Present address: MRC Leukaemia Unit, Hammersmith Hospital, London, W12 0HS.

§ Present address: Department of Medical Oncology, Christie Hospital and Holt Radium Institute, Wilmslow Road, Manchester, M20 9BX.

TABLE I.—*Patient data: ALL* (Total 29; 19M, 10F)

| | Range | Median |
|---|---|---|
| Age (yrs) | 15–68 | 21 |
| Blasts (%) | | |
| Marrow | 77–99 | — |
| Peripheral blood | 0–99 | — |
| WBC ( × 10⁹/l) | 0·8–322·8 | 17·6 |
| Platelets ( × 10⁹/l) | 9–287 | 35 |
| TLI | | |
| All patients | 0·5–67 | 8 |
| Remitters (n = 22) | 0·5–67 | 7 |
| Non-remitters (n = 7) | 4–17 | 11 |

TABLE II.—*Patient data: AML* (Total 172; 96M, 76F)

| | Range | Median |
|---|---|---|
| Age (yrs) | 16–77 | 54 |
| Blasts (%) | | |
| Marrow | 9–99 | — |
| Peripheral blood | 0–99 | — |
| WBC ( × 10⁹/l) | 0·4–500 | 19·6 |
| Platelets ( × 10⁹/l) | 5–398 | 43 |
| TLI | | |
| All patients | 1–86 | 10 |
| Remitters (n = 57) | 1–74 | 9 |
| Non-remitters (n = 115) | 1–86 | 10 |

*Treatment*

A. *Acute myeloblastic leukaemia.*—Between 1970 and 1974, patients were treated with Daunorubicin and cytosine arabinoside (AraC) for remission induction and consolidation. This was followed by monthly maintenance therapy comprising AraC with 6-thioguanine and AraC with Daunorubicin on alternate months.

Between 1974 and 1976, patients were treated with Adriamycin, vincristine, prednisolone and AraC for remission induction and consolidation, and maintenance chemotherapy as above.

From 1970 to 1976 all patients were entered on to trials of "immunotherapy", some receiving no immunotherapy, some BCG alone, and some BCG and allogeneic myeloblasts. None of these manoeuvres influenced the duration of the first complete remission.

B. *Acute lymphoblastic leukaemia.*—All patients were treated with Adriamycin, vincristine, prednisolone and L-asparaginase for remission induction and consolidation, followed by early CNS treatment and continuous maintenance therapy with 6-mercaptopurine, methotrexate and cyclophosphamide.

The details of these treatment programmes have already been published in detail (Crowther *et al.*, 1973; Lister *et al.*, 1978, 1980).

*Thymidine labelling index (TLI)*

The *in vitro* TLI of blast cells in the marrow of all 201 patients was assessed before treatment. Marrow was collected into heparinized Medium 199 (Wellcome) containing 1·25 $\mu$Ci of [$^3$H]dT per ml (sp. act. 5 Ci/mmol) and incubated at 37°C for 30 min. Smears were prepared and after air-drying and methanol fixation they were coated with K5 emulsion (Ilford). Smears were exposed in the dark for 7 days before developing with D19b developer (Kodak) and fixing with Hypam solution (Ilford). Smears were then stained with Leishman's stain. Background was usually less than one grain per cell and labelled cells rarely had less than 100 grains per cell. Depending on the proportion of cells labelled, up to 10,000 cells were counted and the TLI expressed as a percentage. Labelled cells of the erythroid series and labelled lymphocytes were excluded.

### RESULTS

The marrow TLI in 172 patients with AML ranged from 1 to 86% (median 10%) and the TLI in 29 adult patients with ALL ranged from 0·5 to 67% (median 8%). (Fig. 1–5.) There was no significant difference between the TLI in AML and ALL.

The TLI did not correlate with age, sex, the proportion of blasts in the marrow or

FIG. 1.—Relationship between the pre-treatment TLI of marrow blasts and response to initial therapy in AML.

TABLE III.—*Breakdown of labelling-index data: AML*

| | No. of patients | TLI Range | TLI Median | P |
|---|---|---|---|---|
| Total | 172 | 1–86 | 10 | |
| Age (yrs) < 60 | 119 | 1–86 | 9 | |
| > 60 | 53 | 1–53 | 11 | |
| Male | 96 | 1–86 | 10 | 0·13 |
| Female | 76 | 1–56 | 12 | |
| % Blasts in marrow | | | | |
| 1–80 | 89 | 1–86 | 11 | 0·59 |
| 81–99 | 83 | 1–74 | 9 | |
| % Blasts in peripheral blood | | | | |
| 1–70 | 85 | 1–86 | 9 | 0·47 |
| 71–99 | 76 | 1–53 | 11 | |
| Platelets ($\times 10^9$/l) | | | | |
| < 50 | 94 | 1–56 | 10 | 0·25 |
| > 50 | 77 | 1–86 | 10 | |
| Auer rods: present | 39 | 1–56 | 8 | |
| absent | 133 | 1–86 | 10 | |
| Pelger: present | 26 | 2–86 | 12 | |
| absent | 146 | 1–54 | 10 | |
| Abnormal chromosomes | 23 | 2–24 | 9 | |

FIG. 2.—Relationship between the pre-treatment TLI of marrow blasts and the degree of cytological differentiation in AML.

peripheral blood, or the platelet count in AML (Table III). There was no correlation between the TLI and the morphological subdivisions of AML (Fig. 2).

Fifty-seven patients with AML achieved complete remission (33%) but there was no significant difference between the median TLI of the remitters (9%) and of the non-remitters (10%) (Fig. 1). Analysis of the TLI in the 57 patients who achieved complete remission showed no correlation between TLI and remission length (Fig. 3). Of the 29 remission patients with a TLI below 10%, 12 relapsed in less than 8 months, 17 had longer remissions, of whom 2 are still in remission after 56 and 62 months (Fig. 3). Of the 28 patients with a TLI of 10% or more, 16 relapsed in less than 8 months, 12 had longer remissions of whom 3 are still in remission after 49, 64 and 86 months (Fig. 3). (One patient was killed in a road traffic accident whilst still in remission.) These differences were not significant ($P = 0{\cdot}4$). Of the 57 patients who achieved complete remission, 46 were classified as M1 and M2 or M4. Analysis of this group showed that 15/23 patients with a TLI > 10% relapsed in under 8 months, whereas 9/23 patients with a TLI < 10% relapsed in under 8 months. There was, however, no correlation between the length of remission and TLI. Analysis of the survival of the 57 remission patients showed no significant difference between those with a TLI < 10% and those with a TLI of 10% or more ($P = 0{\cdot}7$).

The TLI in all 172 patients with AML showed no correlation with survival. The survival of patients with a TLI of less than 10% and those with a TLI of 10% or more was not significantly different ($P = 0{\cdot}6$) (Fig. 4).

The study included 29 adult patients with ALL, of whom 22 (79%) achieved complete remission. The TLI did not correlate with age, sex, the proportion of blasts in the marrow or peripheral blood, or the platelet count. There was no significant difference between the TLI of remitters and non-remitters (Fig. 5).

FIG. 3.—Relationship between the pre-treatment TLI of marrow blasts and length of first remission in AML (○, still in remission).

FIG. 4.—Comparison of survival curves in AML: patients with a TLI of less than 10% (----) *vs* patients with a TLI of 10% or more (——).

Analysis of TLI in the 22 patients who achieved complete remission showed no correlation between the TLI and remission length (Fig. 6). There was no correlation between the TLI and survival in the 29 patients studied (Fig. 7).

### DISCUSSION

Our earlier conclusion (Crowther *et al.*, 1975) that the pre-treatment TLI correlated with the length of the first remission in AML is no longer tenable. The number of patients in this remission group has increased from 21 to 57. There is still a trend in favour of patients with a TLI below 10% but this is not significant ($P = 0{\cdot}4$) (Fig. 3). Analysis of the 46 patients with M1, M2 and M4 in this remission group shows that there was a tendency for more patients with TLI over 10% to relapse in less than 8 months.

FIG. 5.—Relationship between the pre-treatment TLI of marrow blasts and response to initial therapy in ALL.

However, the TLI did not correlate with remission length. Durie *et al.* (1977) noted a negative correlation between remission length and TLI, but the full data do not seem to have been published.

It can be supposed that the patients seen since 1974 behaved differently to those seen in 1970–1974. Analysis of the 57 remission patients according to treatment shows no correlation between TLI and the induction therapy, maintenance therapy or immunotherapy used. The length of remission in some of the latter patients given more intensive induction chemotherapy was greater (Lister *et al.*, 1980) but there was no correlation with the TLI.

There have been several reports that a higher TLI in patients with AML correlated with the achievement of complete remission (Burke & Owens, 1971; Cheung *et al.*, 1972; Hart *et al.*, 1974; Vogler *et al.*, 1974; Zittoun *et al.*, 1975). Other reports have shown no correlation between the TLI and attainment of complete remission (Amadori *et al.*, 1978; Arlin *et al.*, 1976; Crowther *et al.*, 1975; Durie *et al.*, 1977; Vogler *et al.*, 1976; Wantzin, 1977). It is noteworthy that these papers deal mainly with small numbers of patients and the correlation claimed does not always stand further analysis.

Our data reported here on 172 patients with AML and 29 adult patients with ALL (Fig. 1–7) are in agreement with those of the above authors, who found no correlation between TLI and response to treatment, remission length or survival. A detailed breakdown of our data on TLI in AML is given in Table III. From these data it is clear that the TLI is not a useful parameter in AML. Very little data on adult ALL have been published, but the lack of correlation between the TLI and the response to treatment, remission length or survival in our 29

FIG. 6.—Relationship between the pre-treatment TLI of marrow blasts and length of first remission in ALL ( ○, still in remission).

FIG. 7.—Comparison of survival curves in ALL: patients with a TLI of less than 10% (----) *vs* patients with a TLI of 10% or more (——) $P = 0{\cdot}6$.

cases (Fig. 5, 6, 7) contrasts with the data on childhood ALL (Scarffe *et al.*, 1980).

Acute leukaemia blast cells consist of two distinct populations, dividing and non-dividing, the latter group being the larger. The earliest cells divide, decrease in size and eventually cease to divide. As cells pass through this sequence, the $G_1$ inter-mitotic phase becomes longer. The non-dividing cells are probably in a $G_1$ phase so long that they die before reaching another cell division. The earliest cells have a TLI $> 40\%$, whereas the later cells have a TLI $< 3\%$ (Gavosto *et al.*, 1967; Hillen *et al.*, 1975; Priesler *et al.*, 1977; Sewell, 1967). The observed TLI falls somewhere between the two extremes but is influenced by the relative proportion of non-dividing cells and the rate at which cells die. A TLI of 20% may reflect fewer non-dividing cells rather than greater disease activity than a TLI of 5%. It is interesting to note that although the range of the TLI in our 201 patients was 1–86%, only 23 had a TLI over 20% and only 9 of these had a TLI over 30% (Fig. 1, 5).

Protein synthesis is another factor which must be taken into account. In acute leukaemia there is an accumulation of non-dividing cells which fail to mature. Maturation is essentially the synthesis of enzyme systems. The failure of blast cells to mature may be the result of failure of protein synthesis, for which there is autoradiographic evidence (Gavosto *et al.*, 1960; Sewell, 1972). In acute leukaemia, blast cells readily label with RNA precursors but are largely unlabelled by protein precursors. Further evidence for the failure of these blast cells to make protein has come from studies of ribosomal RNA (rRNA). It has been shown that rRNA in acute-leukaemia blast cells is poorly methylated and largely fails to undergo processing to form the 18S and 28S molecules essential for protein synthesis (Torelli *et al.*, 1970; Seeber *et al.*, 1974). Similar changes in rRNA are seen in growing cells treated with inhibitors of protein synthesis (Craig & Perry, 1970). The failure of protein synthesis in acute-leukaemia blast cells is important as far as the TLI is concerned, because inhibition of protein synthesis has a profound effect on DNA synthesis; entry into S phase is blocked and cells in S cease to synthesize DNA (Brown *et al.*, 1970).

The progression from dividing to nondividing cells, with prolongation of $G_1$ which is seen in acute leukaemia, may thus be secondary to a failure of protein synthesis. Evidence that the failure of protein synthesis may be due to altered transfer RNA continues to accumulate (Sewell, 1967; Weinstein *et al.*, 1971; Harrap, 1978).

Other factors which affect TLI, but about which little is known, are variations in TLI from site to site in the marrow, dilution of the marrow sample with peripheral blood and the ability of blast cells to incorporate dT *via* thymidine kinase.

All these considerations point to a complex situation in which many factors affect the TLI of blast cells in acute leukaemia, and the failure of the TLI to correlate with clinical and haematological parameters is thus not surprising.

Flow cytometry offers a means of obtaining more detailed information about cell kinetics, and there is hope that some of these data will help to predict response to treatment and to determine the value of different drugs (Cullen, 1978; Fulwyler, 1980; Hillen *et al.*, 1975).

This work was supported by the Imperial Cancer Research Fund.

### REFERENCES

AMADORI, S., PETTI, M. C., DE FRANCESCO, A. & 4 others (1978) Prognostic significance of the pretreatment labelling and mitotic indices of marrow blasts in acute nonlymphocytic leukaemia. *Cancer*, **41**, 1154.

ARLIN, Z., GEE, T., DOWLING, M., CAMPBELL, J. & CLARKSON, B. (1976) Significance of Pulse H-Thymidine labelling index (LI) in adult acute myeloid leukaemia (AML). *Proc. Am. Assoc. Cancer Res.*, Abstract 239.

BENNETT, J. M., CATOVSKY, D., DANIEL, M. T. & 4 others (1976) Proposals for the classification of acute leukaemias. *Br. J. Haematol.*, **33**, 451.

BROWN, R. F., UMEDA, T., TAKAI, S. T. & LIEBERMAN, I. (1970) Effect of inhibitors of protein synthesis on DNA formation in liver. *Biochem. Biophys. Acta*, **209**, 49.

BURKE, P. J. & OWENS, A. H. (1971) Attempted recruitment of leukaemic myeloblasts to proliferative activity by sequential drug therapy. *Cancer*, **28**, 830.

CHEUNG, W. H., RAI, K. R. & SAWITSKY, A. (1972) Characteristics of cell proliferation in acute leukaemia. *Cancer Res.*, **32**, 939.

CRAIG, N. C. & PERRY, R. P. (1970) Aberrant intranuclear maturation of abnormal precursors in the absence of protein synthesis. *J. Cell Biol.*, **45**, 554.

CROWTHER, D., BEARD, M. E. J., BATEMAN, C. J. T. & SEWELL, R. L. (1975) Factors influencing prognosis in adults with acute myelogenous leukaemia. *Br. J. Cancer*, **32**, 456.

CROWTHER, D., POWLES, R. L., BATEMAN, C. J. T. & 6 others (1973) Management of adult acute myelogenous leukaemia. *Br. J. Med.*, i, 131.

CULLEN, M. H. (1978) M.D. Thesis, Bristol University.

DURIE, B. G., VAUGHT, L. & SALMON, S. E. (1977) Prognostic significance of tritiated thymidine labelling index (TLI) in multiple myeloma and acute myeloid leukaemia. *Proc. Am. Assoc. Cancer Res.*, Abstract 320.

FULWYLER, M. J. (1980) Flow cytometry and cell sorting. *Blood Cells*, **6**, 173.

GAVOSTO, F., MARAINI, G. & PILERI, A. (1960) Nucleic acids and protein metabolism in acute leukaemia cells. *Blood*, **16**, 1555.

GAVOSTO, F., PILERI, A., GABUTTI, I. & MASERA, P. (1967) Non-self maintaining kinetics of proliferating blasts in human acute leukaemia. *Nature*, **216**, 188.

HARRAP, K. R. (1978) Towards selectivity in cancer chemotherapy: A biochemical overview. *Adv. Enzyme Regul.*, **17**, 457.

HART, J. S., FREIREICH, E. J. & FREI, E. (1974) Prognostic significance of pre-treatment (pre-Rx) proliferative activity in adult leukaemia. *Proc. Am. Assoc. Cancer Res.*, Abstract 290.

HILLEN, H., WESSELS, J. & HAANEN, C. (1975) Bone marrow proliferation patterns in acute myeloblastic leukaemia determined by pulse cytophotometry. *Lancet*, i, 609.

LISTER, T. A., WHITEHOUSE, J. M. A., BEARD, M. E. J. & 8 others (1978) Combination chemotherapy for acute lymphoblastic leukaemia in adults. *Br. Med. J.*, i, 199.

LISTER, T. A., WHITEHOUSE, J. M. A., OLIVER, R. T. D. & 8 others (1980) Chemotherapy and immuno-therapy for acute myelogenous leukaemia. *Cancer*, **46**, 2142.

PREISLER, H. D., WALCZAK, I., RENICK, J. & RUSTUM, Y. M. (1977) Separation of leukaemic cells with proliferative and quiescent subpopulations by centrifugal elutriation. *Cancer Res.*, **37**, 3876.

SCARFFE, J. H., HANN, I. M., EVANS, D. I. K. & 4 others (1980) Relationship between the pretreatment proliferative activity of marrow blast cells and prognosis of acute lymphoblastic leukaemia of childhood. *Br. J. Cancer*, **41**, 764.

SEEBER, S., KADING, J., BRUCKSH, K. P. & SCHMIDT, C. G. (1974) Defective rRNA synthesis in human leukaemic blast cells. *Nature*, **248**, 673.

SEWELL, R. L. (1967) The cytology and cytochemistry of acute leukaemias: A critical review. *J. Med. Lab. Technol.*, **24**, 1.

SEWELL, R. L. (1972) *Malignant Blood Diseases.* London: Baillere Tindall. p. 31.

TORELLI, U. L., TORELLI, G. M., ANDREOLI, A. & MAURI, C. (1970) Partial failure of methylation and cleavage of 45S RNA in the blast cells of acute leukaemia. *Nature*, **226**, 1163.

VOGLER, W. R., COOPER, L. E. & GROTH, D. P. (1974) Correlation of cytosine arabinoside-induced increment in growth function of leukaemic blast cells with clinical response. *Cancer*, **33**, 603.

VOGLER, W. R., KREMER, W. B., KNOSPE, W. H., OMURA, G. A. & TORNYOS, K. (1976) Synchronization with phase specific agents in leukaemia and correlation with clinical response to chemotherapy. *Cancer Treat. Rep.*, **60**, 1845.

WANTZIN, G. L. (1977) Nuclear labelling of leukaemic blast cells with tritiated thymidine triphosphate in 35 patients with acute leukaemia. *Br. J. Haematol.*, **37**, 475.

WEINSTEIN, I. B., GRUNBERGER, D., FUJIMURA, S. & FINK, L. M. (1971) Chemical carcinogens and RNA. *Cancer Res.*, **31**, 651.

ZITTOUN, R., BOUCHARD, M., FACQUET-DANIS, J., PERCIE-DU-SERT, M. & BOUSSER, J. (1975) Prediction of the response to chemotherapy in acute leukaemia. *Cancer*, **35**, 507.